# Translational cardiac stem cell therapy: advancing from first-generation to next-generation cell types

**DOI:** 10.1038/s41536-017-0024-1

**Published:** 2017-06-13

**Authors:** Elena Cambria, Francesco S. Pasqualini, Petra Wolint, Julia Günter, Julia Steiger, Annina Bopp, Simon P. Hoerstrup, Maximilian Y. Emmert

**Affiliations:** 1 Institute for Regenerative Medicine, University of Zurich, Zurich, 8044 Switzerland; 2 Division of Surgical Research, University Hospital of Zurich, Zurich, 8091 Switzerland; 3 Heart Center Zurich, University Hospital of Zurich, Zurich, Switzerland; 4 Wyss Translational Center Zurich, Zurich, Switzerland

## Abstract

Acute myocardial infarction and chronic heart failure rank among the major causes of morbidity and mortality worldwide. Except for heart transplantation, current therapy options only treat the symptoms but do not cure the disease. Stem cell-based therapies represent a possible paradigm shift for cardiac repair. However, most of the first-generation approaches displayed heterogeneous clinical outcomes regarding efficacy. Stemming from the desire to closely match the target organ, second-generation cell types were introduced and rapidly moved from bench to bedside. Unfortunately, debates remain around the benefit of stem cell therapy, optimal trial design parameters, and the ideal cell type. Aiming at highlighting controversies, this article provides a critical overview of the translation of first-generation and second-generation cell types. It further emphasizes the importance of understanding the mechanisms of cardiac repair and the lessons learned from first-generation trials, in order to improve cell-based therapies and to potentially finally implement cell-free therapies.

## Introduction

Myocardial infarction (MI) mortality decrease^1^ has contributed with an aging population to the rise of heart failure (HF) incidence.^1^ After MI, cardiomyocyte death triggers wall thinning, ventricular dilatation, and fibrosis that can cause left ventricular (LV) dysfunction and HF.^2^ HF counts 30 million patients^1^ and a ~50% death rate within 5 years post diagnosis.^3^ Pharmacological therapies and revascularization techniques (e.g., percutaneous coronary intervention (PCI) and coronary artery bypass grafting (CABG)) have improved patient survival and quality of life, but cannot stop or reverse HF. The heart can ultimately be supported by left ventricular assist devices or replaced by transplantation, but organ shortage, high costs, and complex postoperative management limit these strategies. Hence, novel curative treatments are needed.

Stem cell therapy has been proposed for heart repair and regeneration. The exact mechanisms of cardiac repair by transplanted cells are merely unknown. Two main hypotheses exist: (1) direct cardiomyogenic/vasculogenic differentiation, and (2) indirect stimulation of the reparative response through paracrine effects.^4^

Different cell types are under evaluation regarding their regenerative potential. First-generation cell types including skeletal myoblasts (SMs), bone marrow mononuclear cells (BMMNCs), hematopoietic stem cells (HSCs), endothelial progenitor cells (EPCs), and mesenchymal stem cells (MSCs) were initially introduced. Despite promising preclinical studies, first-generation approaches displayed heterogeneous clinical outcomes.^4, 5^ Variations between trials may be attributed to differences in design (cell preparation, delivery route, timing, dose, endpoints, and follow-up (FU) methods). Well-conducted recent meta-analyses reviewed the efficacy of (mostly first-generation) cell-based approaches and came to divergent conclusions.^6–8^

**Fig. 1:**
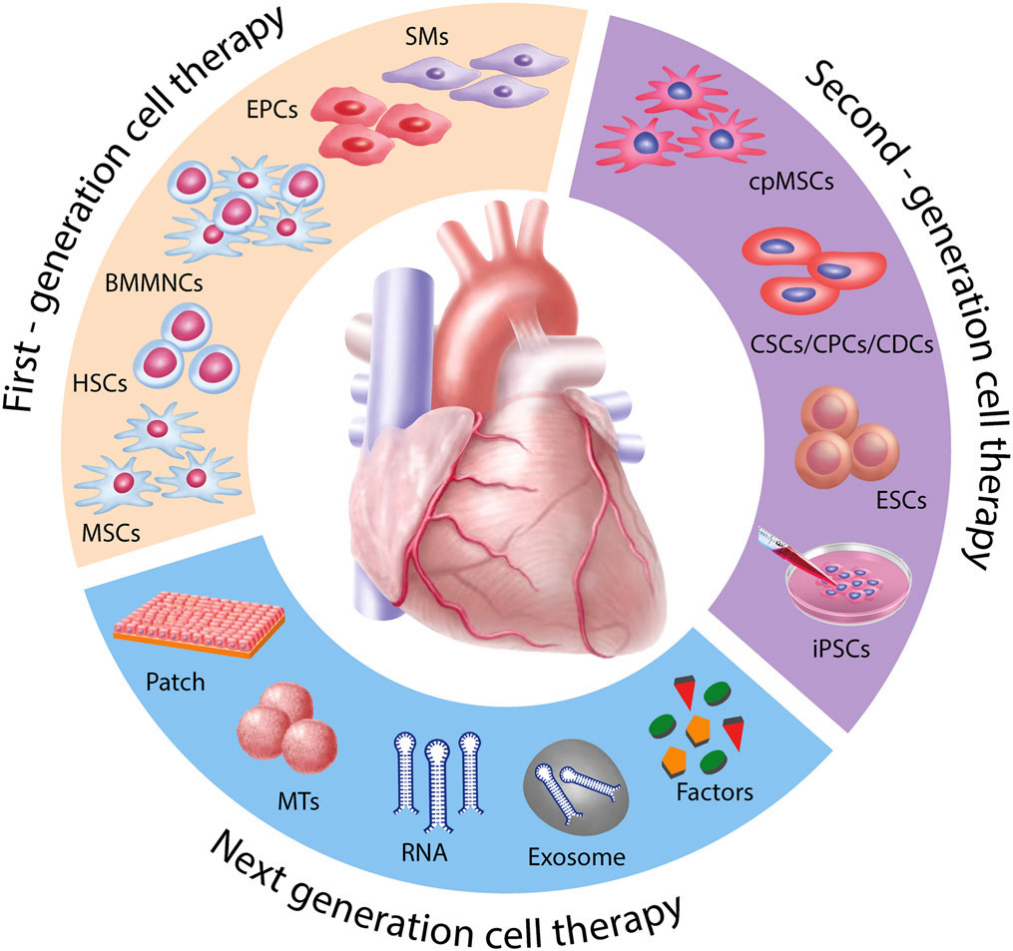
Evolution of translational cardiac regenerative therapies. First-generation cell types such as SMs, BMMNCs, HSCs, EPCs, and MSCs demonstrated feasibility and safety with, however, heterogeneous outcomes and limited efficacy in the clinical setting. In order to better match the target organ, second-generation cell therapies propose the use of cpMSCs, CSCs/CPCs, and CDCs, and pluripotent stem cells such as ESCs and iPSCs. Next-generation therapies for cardiac repair are directed toward cell enhancement (e.g., biomaterials, 3D cell constructs, cytokines, miRNAs) and cell-free concepts (e.g., growth factors, non-coding RNAs, extracellular vesicles, and direct reprograming)

Nevertheless, the field partially switched to second-generation cell types including lineage-guided cardiopoietic cells, cardiac stem/progenitor cells (CSCs/CPCs), and pluripotent stem cells (Fig. 1).

This article provides a critical overview of the translation of first-generation and second-generation cell types with a particular focus on controversies and debates. It also sheds light on the importance of understanding the mechanisms of cardiac repair and the lessons learned from first-generation trials, in order to improve cell-based therapies and to potentially finally implement cell-free therapies.

## First-generation cell types

### Skeletal myoblasts

With the goal of remuscularizing the injured heart and based on the inference that force-generating cells would function in the cardiac milieu and increase cardiac contractility, SMs figured among the first cell types to be tested. They can be obtained in high number from autologous skeletal muscle satellite cells by expansion in vitro, can be activated in response to muscle damage in vivo, and are resistant to ischemia.^9^

#### SMs in preclinical trials

Initial studies in small and large animals were encouraging, with SMs participating at heart muscle formation.^10, 11^ However, SMs were shown to not electrophysiological couple to native cardiomyocytes in rodents.^12, 13^ Indeed, N-cadherin and connexin-43 expression was downregulated after transplantation.^12^ SMs did not differentiate into cardiomyocytes in rodents,^14^ but could surprisingly differentiate into myotubes in sheep,^15^ although these findings could not be replicated. Small and large animal trials were nonetheless further conducted and displayed an improvement of LV function.^15–17^ The involved mechanisms were, however, not understood.

#### SMs in clinical trials

Despite the mixed outcomes in preclinical trials, SMs were rapidly translated into the clinics with phase-I trials in both MI and HF.^18–23^ Although the transplantation of autologous SMs displayed an arrhythmogenic potential in a phase-I trial of severe ischemic cardiomyopathy (ICM),^24^ SMs were further implanted in the randomized phase-II MAGIC study (97 patients with severe LV dysfunction).^25^ However, an increased risk of ventricular arrhythmias potentially due to missing junctional proteins^26^ stopped SMs investigation. The risk of ventricular arrhythmias is relevant now that pluripotent cell-derived cardiomyocytes aim at re-attempting heart remuscularization.

### Bone marrow (BM)-derived cells

Moving away from remuscularization, strategies using stem cells aimed at direct/indirect regeneration. The main stem cell source for these early studies was the BM. Investigated cell types were mostly BMMNCs and their subpopulations including HSCs. Blood-circulating EPCs, probably originating from the BM, were also adopted. BMMNCs have constituted a most often used cell source due to their safety, high availability,^27^ and facile isolation. HSCs can be isolated via surface markers such as CD34 and CD133. EPCs can simply be harvested from a blood sample.

#### BM-derived cells in preclinical trials

BMMNCs were among the first cells to be tested in large animals despite inconsistent reports on their mechanism of action. Differentiation into cardiomyocytes was first observed in rodents,^28, 29^ but was criticized later.^30^ Large animal preclinical studies yielded promising results with, however, mixed outcomes.^31–34^ Regarding HSCs, one of the few existing large animal studies found no evidence of myocardial differentiation of CD34^+^ HSCs, but showed increased angiogenesis/vasculogenesis, potentially due to paracrine effects on the host vasculature.^35^ Few large animal and clinical studies were conducted with EPCs and their results were mixed.^36–38^

#### BM-derived cells in clinical trials

Although BM-derived cells showed encouraging preliminary results, efficacy outcomes were heterogeneous in clinics.^4^ While some clinical trials showed modest but significant improvement of cardiac function,^39–41^ others did not find significant beneficial effects of cell therapy.^42–45^

In acute MI, first-generation cell-based clinical trials were all performed using intracoronary delivery of autologous BMMNCs.^46–50^ HEBE^43, 51^ and TOPCARE-AMI^52^ also investigated other progenitor cells. While certain trials (TOPCARE-AMI,^52^ REPAIR-AMI,^53–55^ BOOST,^56, 57^ and FINCELL)^41^ have shown improvement in LV ejection fraction (LVEF) in cell-treated groups compared to controls, others did not display any significant change (ASTAMI,^58^ BONAMI,^59^ Leuven-AMI,^60^ and HEBE)^43, 51^ at early FU. At a long-term 5-year FU, the beneficial effect of cell therapy persisted in TOPCARE-AMI^61^ but not in BOOST,^62^ which was the first published clinical trial to assess BMMNC injection compared to controls in 60 patients. The REPAIR-AMI trial (NCT00279175) was the largest European cell therapy study of autologous BM-derived progenitor cells with 204 patients. Patients underwent BM aspiration 3–6 days after successful PCI following MI. Either BMMNCs or placebo were infused via intracoronary delivery the following day. Quantitative LV angiography was performed to measure the change in global LVEF between baseline and 4-month FU. At 4 months, the cell-treated group displayed a significantly higher increase in LVEF compared to placebo.^53^ This improvement in LVEF was sustained at 2-year FU,^55^ thus contradicting the results of BOOST, where this effect was lost after 18 months.^57^ The HEBE trial is another large European trial where 200 patients were randomized to treatment with BMMNCs, peripheral blood mononuclear cells (PBMCs) or placebo. No difference between cell-treated groups and control group was shown at 4 months by cardiac MRI (cMRI).^43^ The 5-year FU after AMI displayed a significantly higher frequency of major clinical cardiovascular adverse events in the PBMC group compared to placebo.^51^

Similar results to the early FU of HEBE are shown by the SWISS-AMI trial (NCT00355186), where 192 MI patients were assigned to one control and two BMMNC treatment groups. BMMNC groups received intracoronary administration of autologous BMMNC at 5–7 days or 3–4 weeks after MI.^63^ Cell infusion at either early or late time points did not significantly improve LV function at 4 months as measured by cMRI.^64^ At 12 months, BMMNC treatment did not improve LV function compared to control. An important drop-out rate limited the results.^65^

To put an end to the ongoing controversies and to further elucidate the clinical value of intracoronary-delivered autologous BMMNCs, the ongoing BAMI trial (NCT01569178) aims to examine the time from randomization to death for an average time frame of 3 years. This multicenter, randomized, controlled, phase-III study is investigating safety and reduction of all-cause mortality in patients with reduced LV function (LVEF ≤ 45%) after successful PCI following MI. About 3000 patients will be enrolled. While BAMI is expected to either definitely confirm BM cell therapy efficacy or disprove it, the trial is criticized because it may not teach anything new about the mechanism of either outcome.

To tackle the problem of the optimal timing for cell administration, two phase-II trials were performed in MI patients. In TIME (NCT00684021) and LateTIME (NCT00684060), 150 × 10^6^ cells were delivered at 3/7 days^66^ and 2–3 weeks^67^ post PCI, respectively. These randomized, double-blind, placebo-controlled trials involved 120^66^ and 87^67^ patients, respectively. At 6 months, no significant increase in LVEF was observed in the BMMNC group compared to placebo in both trials.^68, 69^ Overall, TIME and LateTIME did not find significant benefit of early vs. late BMMNC treatment.^68, 69^

BM-derived cells have also been investigated in HF,^70–77^ yielding mixed outcomes as in MI. The phase-II FOCUS-CCTRN trial (NCT00824005) studied the effect of transendocardial NOGA® delivery of 100 × 10^6^ autologous BMMNCs or placebo to 92 patients. BMMNCs did not significantly improve maximal oxygen consumption, LV end-systolic volume (ESV), or reversibility on single-photon emission computed tomography compared to placebo after 6 months.^44^ These results contradict the positive outcomes previously obtained by the TOPCARE-CHD trial, where intracoronary BMMNC-treated HF patients showed a 2.9% increase in LVEF at 3 months compared to baseline.^40, 78^ All together, the outcomes of therapy using BM-derived and progenitor cells were heterogeneous and rather disappointing whether it was in MI or HF. Possible reasons may be low cell engraftment and limited differentiation potential,^5^ suggesting that the modest improvements yielded by cell therapy may be due to paracrine mechanisms rather than direct regeneration.

### Mesenchymal stem cells

MSCs are multipotent, plastic-adherent stromal cells that can differentiate into different cell types including adipocytes, chondrocytes, and osteocytes.^79^ Although controversially discussed, differentiation into cardiomyocytes was shown in experimental studies.^80^ MSCs may be found in all postnatal organs and the presence of MSCs was shown in mouse hearts.^81, 82^ Cells positive for W8B2 antigen highly expressing mesenchymal markers have recently been discovered in the human heart.^83^ Human MSCs have most often been isolated from the BM (BM-MSCs) but can also be obtained from adipose tissue, synovial tissue, umbilical cord, and peripheral blood.^80^ Besides autologous usages, MSCs were also considered in allogeneic therapies due to their high expansion rate and immunomodulatory properties. Interestingly, MSCs were originally considered immuno-privileged, due to their cytokine secretion and surface antigen expression,^80^ but conflicting reports from preclinical studies have questioned this property.^84^ Allogeneic MSCs may lose their immune privilege upon differentiation,^85^ thus leading to earlier clearance compared to autologous MSCs.^86^

#### MSCs in preclinical trials

MSCs have extensively been investigated in vivo.^87–91^ Preclinical trials have shown that adipose tissue-derived MSCs represent an auspicious cell source with therapeutic potential for cardiac repair.^92, 93^ BM-derived MSCs were promising in numerous preclinical trials. Regarding efficacy, MSCs were administered to pigs with encouraging outcomes.^94–97^

#### MSCs in clinical trials

Approaches using MSCs are studied with promising results,^74, 98–102^ but their efficacy needs to be further validated. Preliminary studies on ICM were performed in the POSEIDON trial (NCT01087996). This phase-I/II randomized non-controlled pilot study compared the safety and efficacy of transendocardial delivery of autologous vs. allogeneic BM-MSCs in 30 patients. Three different cell doses (20, 100, and 200 million cells) were tested in both treatment groups. Surprisingly, the lowest dose yielded the best outcomes in terms of LV volumes and LVEF. Moreover, despite its small size, POSEIDON has given preliminary evidence of comparable safety and efficacy between autologous and allogeneic MSCs.^103, 104^ Larger controlled trials are needed to further investigate MSC efficacy.

### Meta-analyses of cell therapy in MI and HF

#### Meta-analyses of preclinical trials

A compelling meta-analysis of large animal models of ischemic heart disease (IHD) analyzed 52 studies and 888 animals. In addition to confirming the safety of cell therapy, a difference of 7.5% in LVEF at FU compared to controls was found.^105^ Although BMMNCs and MSCs were the most used cell types, trends suggested that BMMNCs were less effective than other cell types.

A new meta-analysis of large animal studies (82 studies with 1415 animals) in the context of autologous and allogeneic cell therapy for IHD^106^ showed a significant difference of 8.3% in LVEF and a significant decrease in end-diastolic volume (EDV) between treated and control animals. Similar differences in LVEF were observed for both autologous and allogeneic therapies.

#### Controversies in meta-analyses of clinical trials

Several meta-analyses have assessed cell therapy in clinical trials. In order to improve the analysis of the safety and efficacy of cell therapy in MI, the first multinational database of individual patient data (IPD) (ACCRUE, NCT01098591) was established.^6^ ACCRUE contains unbiased data with uniform clinical definitions and parameters. This allows the examination of specific patient subgroups and the identification of predictive factors for the improvement of cell therapy. One thousand two hundred fifty-two IPD from 12 randomized trials of intracoronary cell therapy after MI were analyzed. Although the results showed cell therapy safety, they did not display any efficacy compared to controls and no predictive factors could be identified. Timing/dose of cell therapy and baseline EF did not influence the results. Although the study investigated mainly first-generation cell types, the main limitation was the variety in cell types. The database is still growing but cannot replace large randomized trials, such as the current BAMI trial.^6^

Interestingly, while several publication-based meta-analyses report an effect of BM-derived cell therapy,^107, 108^ another recent meta-analysis of cell therapy in MI has further showed no difference between cell-treated and control-groups when the LV parameters were assessed by cMRI.^8, 109^ Indeed, the endpoints and the FU method can also influence the outcomes. The DAMASCENE study has evidenced that the change in EF might be a problematic endpoint, as a higher number of discrepancies in trial reporting is associated with a better change in EF. It was indeed found that factual discrepancies are present in autologous BM cells trials and that trials having >30 discrepancies showed a mean EF effect size of 7.7%, while trials having no discrepancies (only 5 trials over 49 examined trials) showed a mean EF effect size of −0.4%.^110^ However, the DAMASCENE study has also been challenged as misleading^111^ and a meta-analysis has shown significant cell therapy efficacy when the discrepant trials were excluded.^112^

In HF, a meta-analysis including 31 randomized controlled trials (RCTs) with 1521 patients assessed the safety and efficacy of autologous cell therapy.^7^ A comparison was performed between cell treatment and placebo/controls. Cell therapy was associated with a significant decrease in mortality and rehospitalization during long-term FU. Furthermore, cell treatment improved LVEF significantly but modestly. HF symptoms, exercise capacity, and quality of life ameliorated significantly. Nevertheless, only half of the examined trials reported blinding and half did not report methods of allocation concealment, thus considerably increasing the performance/selection bias.^7^ The difference between cell treatment and control groups was indeed eliminated when only double-blind trials were included.^7^ Therefore, further larger RCTs are necessary to confirm clinical long-term efficacy in HF.

## Second-generation cell types

Motivated by the inconsistencies of first-generation cell types, the field has shifted toward the use of other cell types. Second-generation therapies aim at orienting non-resident stem cells, such as MSCs and pluripotent stem cells, toward cardiac differentiation. CSCs/CPCs may further better match the target organ, as they are derived directly from the heart.

Only few experimental studies compared first-generation and second-generation cell types. They found that cardiac-committed cells displayed an improved therapeutic effect as assessed by improved engraftment, cardiac function, angiogenesis, and scar size.^113–117^

### Cardiopoietic MSCs (cpMSCs)

Guided cardiopoiesis using cardiogenic growth factors priming has been introduced and advanced into the clinics. One of the cell sources used for guided cardiopoiesis is autologous BM-MSCs. Cytosolic expression of cardiac transcription factors is induced by simultaneous activation with TGF-β, BMP-4, and Activin-A along with retinoic acid, while their nuclear translocation is prompted by IGF-1 and IL-6.^1^ FGF-2 and thrombin are further used to maintain cell cycle activity.^1^

#### cpMSCs in preclinical trials

The use of cpMSCs in a murine model of chronic ICM has shown therapeutic benefit.^118^ Large animal trials were missing so far. A first report of safety and efficacy of intramyocardial delivery of human cpMSCs into immunosuppressed pigs with post-infarction LV dysfunction has recently shown promising results,^119^ including higher EF and reduced infarct size compared to controls. The low cell retention suggested the involvement of paracrine mechanisms in neo-angiogenesis and recruitment of endogenous progenitors. These findings need to be validated in long-term studies.

#### cpMSCs in clinical trials

Despite the absence of large animal studies, cpMSCs were rapidly introduced into the clinics. The multicenter randomized phase-II C-CURE trial (NCT00810238) investigated the transendocardial injection of cardiopoietic BM-MSCs. A non-significant increase in LVEF compared to baseline was shown in the cell treatment group but not in the control group. Besides indicating clinical feasibility/safety at 2-year FU, the trial claims to display signs of efficacy.^120^ Of note, two phases were included in the initial design of C-CURE: a safety/feasibility phase and a potential later efficacy phase. However, the trial was limited to the first phase based on advice from regulatory authorities.^121^

Based on C-CURE data, a progressive translation into the two phase-III CHART trials was initiated. CHART-1 (NCT01768702) is an ongoing controlled multicenter, randomized clinical trial, evaluating cpMSCs in ischemic HF. The trial randomized 315 patients and 271 patients were analyzed for efficacy (120 received cpMSCs and 151 sham control). However, the CHART-1 trial failed to meet its primary efficacy endpoint at 39 weeks.^122^ The authors identified post hoc a responsive patient subgroup based on baseline HF severity (LV EDV of 200–370 ml). The CHART-2 trial (NCT02317458) will target these type of patients. This further poses the question of which cell type to use depending on patient/pathology.

### Cardiac stem/progenitor cells

CSCs/CPCs are derived directly from biopsies of the target organ, and therefore supposedly ensure a perfect match.^4^ CSCs/CPCs are multipotent, clonogenic, and express stem cell markers such as Sca1^123, 124^ and c-kit.^124, 125^ Sca1^+^ CPC exosomes can inhibit cardiomyocytes apoptosis.^126^ Of note, there is a lack of an agreed Sca1 equivalent in humans. Several studies have associated c-kit with cardiomyocyte biology.^127–131^ Although it was shown in rodents that c-kit^+^ CSCs/CPCs could differentiate into cardiomyocytes,^125, 132, 133^ this was challenged by lineage-tracing analysis studies that suggested that this phenomenon occurs at a purported functionally insignificant rate.^134–137^

Cells derived from cardiac explants can form cardiospheres, which can be dissociated to yield cardiosphere-derived cells (CDCs). All these cell types are thought to possess enhanced regeneration capacity through the stimulation of endogenous cardiac cells and/or paracrine mechanisms. Of interest is also the combination of CSCs/CPCs with MSCs to achieve a synergistic effect.^138, 139^

#### CSCs/CPCs in preclinical trials

CSCs/CPCs are studied in the preclinical setting.^140–142^ Interestingly, a recent report showed that overexpression of Pim1 kinase enhanced the cardiac repair potential of human c-kit^+^ CSCs transplanted into an MI swine model.^143^ CDCs were administered to pigs with encouraging efficacy outcomes.^144, 145^ Allogeneic CDCs were transplanted via intracoronary delivery at escalating doses between 5 and 10 million cells in an MI pig model.^146^ This study showed safety/feasibility and significant cardioprotection with reduced infarct size, microvascular obstruction, and adverse remodeling compared to controls.

Recently, a compelling meta-analysis of CSC therapy in preclinical MI studies has showed an LVEF improvement of 10.7% in cell-treated animals compared to controls.^147^

Interestingly, MSCs/CSCs combination has yielded encouraging results.^138, 139^ Transendocardially delivered MSCs and c-kit^+^ CSCs have showed positive synergistic effects in a swine model after ischemia/reperfusion injury.^139^ However, these results await further confirmation from additional studies.

#### CSCs/CPCs in clinical trials

New controversies have also emerged with the second-generation era. Two major phase-I trials assessed cardiac-derived cells for the first time in the clinics. The randomized SCIPIO trial investigated the safety and efficacy of intracoronary c-kit^+^ CSC therapy in 33 ICM patients (20 treated and 13 controls). About 113 days after CABG, 1 × 10^6^ autologous cells were injected.^148^ The cell-treated group displayed a significant increase in LVEF at 4 and 12 months.^149^ After CSC injection, decreases in infarct size of 22.7 and 30.2% were measured at 4 and 12 months, respectively.^148, 149^ Nevertheless, this trial is subject to an expression of concern by *The Lancet*.^150^

The CADUCEUS trial (NCT00893360) is a randomized study of the safety and preliminary efficacy of intracoronary delivery of autologous CDCs in patients with LV dysfunction after MI. CDCs were applied in 17 patients 1.5–3 months after MI with a varying dose of 12.5–25 × 10^6^ cells. Eight patients were assessed as standard care patients.^151, 152^ No tumor formation, major adverse cardiac events, or deaths were observed after 6 months. While CDC treatment resulted in favorable structural changes (scar mass, viable heart mass, regional contractility, and systolic wall thickening) compared to controls, no changes in EDV, ESV, and LVEF were observed at 6 months.^151^ At 1-year FU, signs of efficacy were displayed, as measured by reduced scar size and improvement in regional function compared to controls.^152^ Although CADUCEUS is a well-performed study, its small size prevents judging efficacy.

Further ongoing studies are evaluating the safety and efficacy of CSCs/CPCs. The first study to address the safety and efficacy of allogeneic CDC therapy in phase-II is the ALLSTAR trial (NCT01458405). Enrollment is completed according to the sponsor. Allogeneic CSCs are also currently tested in the CAREMI trial (NCT02439398).

Another interesting hybrid approach is presented by the preliminary phase-I ALCADIA trial (NCT00981006) with autologous CDCs and controlled release of basic fibroblast growth factor to treat ICM. Results have shown increased LVEF and decreased scar size 6 months after treatment.^153, 154^ However, ALCADIA is a small study (*n* = 6) without control group and further trials are needed.

To further advance the preclinical CSCs/MSCs combinatorial approach, the phase-II, randomized, placebo-controlled CONCERT-HF trial (NCT02501811) is recruiting participants to investigate the feasibility/safety and effect of autologous BM-MSCs and c-kit^+^ CSCs delivered by transendocardial injection in ICM subjects.

Although initial clinical results from studies investigating CSCs/CPCs are promising, demonstrating feasibility/safety and signs of efficacy, these cell types must be further assessed in larger cohorts and their mechanisms of cardiac repair must be fully elucidated.

### Pluripotent stem cells

Pluripotent stem cells, including embryonic stem cells (ESCs) and induced pluripotent stem cells (iPSCs), constitute another source for guided cardiac differentiation. ESCs can differentiate into any cell type found in the adult organism. Human ESC-derived cardiomyocytes express cardiac transcription factors and display adult cardiomyocyte phenotype and beating activity in vitro.^155^ However, common concerns include ethical issues and safety, since residual undifferentiated cells could induce teratoma formation.

iPSCs constitute a potential alternative to ESCs as they display similar characteristics while avoiding the ethical debate. iPSCs are obtained from adult somatic cells by forcing the re-expression of key reprogramming genes. However, many questions and safety issues such as tumor formation remain to be clarified.

#### Pluripotent stem cells in preclinical trials

Preclinical studies with pluripotent stem cells yielded mixed results depending on the animal model. Differences between rodents and large animals were repeatedly noted. Cardiac-committed mouse ESCs were successfully implanted into sheep, resulting in improved LV function.^156^ Similar results have also been observed in rodents, but with teratoma formation.^155^ Human ESC-derived cardiomyocytes were implanted in macaques.^157^ The infarcted heart was “remuscularized” and cardiomyocytes underwent progressive but incomplete maturation over 3 months. The grafts were vascularized and electrically coupled. Although the electrical coupling was enhanced compared to studies of the same group in rodents, non-fatal ventricular arrhythmias not observed in rodents could be detected in monkeys.^157^ It should be noted that the findings of this study have been challenged in the literature.^158^

In several preclinical trials, human ESC-derived CPCs have also been transplanted into small and large animal models of MI, showing improved cardiac function. Cell embedding into a fibrin patch has further improved cell engraftment and efficacy, thus leading to clinical trials.^159^

Cell sheets made of human iPSC-derived cardiomyocytes were delivered into a swine ICM model.^160^ Despite low long-term cell survival, no teratoma was observed and cardiac function was improved. Recently, human iPSCs have been differentiated into the three cardiac lineages. Their transplantation in a pig MI model showed cell engraftment and improved cardiac function without ventricular arrhythmias.^161^ Issues such as rejection and teratoma formation need to be further addressed before advancement into clinics.

#### Pluripotent stem cells in clinical trials

Witnessed with both apprehension and curiosity by the scientific community, human ESC-based therapy has also recently advanced into the clinics. The proof-of-concept ESCORT trial (NCT02057900) is testing ESC-derived CPCs (CD15^+^ Isl-1^+^ progenitors) embedded into a fibrin scaffold. The patch was delivered for the first time into a patient with advanced ischemic HF.^162^ While preliminary outcomes are promising and show the feasibility of producing clinical-grade ESC-derived CPCs, the forthcoming results of the study are necessary to draw a conclusion.^162^

## Cell enhancement and cell-free approaches: the next generation?

### Cell enhancement approaches

Several strategies are investigated to ameliorate the poor performance of transplanted cells. They mainly consist in improving cell retention, survival, coupling, and differentiation. To improve cell retention, scaffold-based and scaffold-free approaches can be used. Scaffolds for cardiac cell therapy include decellularized matrices, injectable biomaterials, and cardiac patches made of synthetic or natural hydrogels.^163, 164^ Scaffold-free tissue-based constructs such as cell sheets and microtissues also exist.^165–167^ Bispecific antibodies can also be used to link cells to the injured heart.^168^ The CELLWAVE trial (NCT00326989) used shock-wave therapy to promote homing of BMMNCs in HF patients.^169^ Survival and angiogenesis can be improved by using pro-survival and angiogenic cytokines or by modification of specific genes.^170, 171^ Overexpression of N-cadherin and connexin-43 could improve coupling. Cells can also be pre-conditioned in hypoxic conditions^172^ and differentiation can be enhanced with microRNAs (miRNAs).^173, 174^

### Cell-free approaches

Based on the hypothesis that cell therapy mainly functions through paracrine mechanisms, new strategies propose to skip the cells and only use the supposedly paracrine factors. These approaches mainly include the administration/regulation of growth factors and non-coding RNAs. Following the rationale of in situ modification, direct reprogramming aims to convert scar fibroblasts into cardiomyocyte-like cells.

#### Administration/regulation of growth factors

Examples of investigated growth factors are the vascular endothelial growth factor (VEGF), the granulocyte-colony stimulating factor (G-CSF), and erythropoietin (Epo). VEGF gene therapy failed to improve perfusion of ischemic myocardium in the NORTHERN clinical trial.^175^ G-CSF did not display significant improvement in myocardial function compared to placebo in the clinics.^176–178^ While administration of Epo displayed encouraging results with preservation of cardiac function in infarcted mice,^179^ the phase-III REVIVAL-3 trial (NCT00390832) showed no improvement in LVEF or infarct size compared to placebo at 6 month FU.^180^ A lack of reduction in infarct size was also documented by other clinical studies with shorter FU times.^181, 182^ The poor outcomes of growth factor-based approaches may be due to inappropriate dosages and/or the lack of organ selectivity, among others.

#### Administration/regulation of non-coding RNAs

Non-coding RNAs include miRNAs and long non-coding RNAs. They may represent possible therapeutic targets due to their abundance in the cardiovascular system and their potential function in heart physiology and disease.^183^ miRNAs have been mainly investigated in mice but also in large animals. In a porcine MI model, local and selective inhibition of miR-92a resulted in enhanced angiogenesis and prevention of adverse remodeling.^184^ miRNAs are found at the intracellular level but also in extracellular vesicles, such as exosomes. Exosomes also contain mRNAs, proteins and lipids, and are thought to play a role in cell–cell communication and in cardiovascular physiology.^185^ They are currently investigated as diagnostic markers and their roles may also encompass cardioprotection.^186^ Human CPC-derived extracellular vesicles have displayed a decrease in cardiomyocyte apoptosis and an increase in angiogenesis and LVEF in acute MI rats.^187^ Following positive results in vitro and in rodents,^188, 189^ CDC-derived exosomes were shown to decrease infarct size and preserve LVEF in a recent preclinical study in acute and chronic porcine MI.^190^ Interestingly, this effect was observed in acute MI with intramyocardial but not with intracoronary injection. Mouse ESC-derived exosomes displayed enhanced cardiac function and repair in infarcted mice.^191^ Nevertheless, it is challenging to separate the relative contributions of regeneration vs. salvage of existing myocardial tissue.

Extracellular vesicles have raised a great interest. However, several open issues remain to be addressed before they can fully supplant cell-based therapies. For instance, the type of donor cells, the type and size of vesicles, their content and their potential immunogenicity need to be investigated in further detail.

#### Direct reprogramming

To achieve direct reprogramming, a specific cocktail of transcription factors^192–194^ or miRNAs^195^ can be used. A recent report has shown the feasibility of direct reprogramming of human fibroblasts into cardiomyocyte-like cells using only small molecules.^196^ Direct reprogramming of murine fibroblasts into cardiomyocyte-like cells was shown in vitro and in vivo^192–194^ and has opened the way to large animal studies. The field of direct reprogramming is still at its infancy and has to cope with several issues before it can reach clinical translation. Vectors need to safely and efficiently transfect the heart cells without triggering the immune response, which could clear the vectors and the transfected cells. Only fibroblasts and no other neighboring cells should be targeted. Moreover, direct reprogramming approaches need to be tested with human cells and large animal models before they can reach the clinics.

## Conclusion

Cell therapy holds potential to tackle MI and HF. Issues such as the cell type, cell number, delivery route, timing, FU periods, and endpoints remain unsolved. The field has rapidly evolved to address in particular the ideal cell type. The first attempts of heart remuscularization with SMs were abandoned due to ventricular arrhythmias. Then, the rationale of direct/indirect regeneration by stem cells was adopted. While first-generation cells such as BM-derived cells and MSCs gave overall promising results in preclinical studies, they yielded heterogeneous efficacy outcomes in the clinics. The reasons include differences in trial design and a too rapid translation despite a lack of understanding of the biological mechanisms. Prominent meta-analyses have reached contradictory results about cell therapy efficacy. Large clinical trials such as BAMI will hopefully settle the discussion. Motivated by the desire to match the target organ, second-generation approaches currently investigate cpMSCs, CSCs/CPCs, and pluripotent stem cells. While encouraging results were displayed in both the preclinical and clinical settings, controversies exist. Notably, cpMSCs entered the clinics without previous large animal trials and CHART-1, which did not meet its primary efficacy endpoint, was initiated without a clear evidence of efficacy in a phase-II trial. CSCs/CPCs are promising but their mode of action should be further investigated. Pluripotent stem cells have made their entrance into the clinics despite a lack of uniformity between small and large animal studies. Their advancement is now followed with both interest and apprehension. And yet, it is crucial to learn from first-generation trials and to gain a better understanding of the mode of action of transplanted cells. Future preclinical trials should not only test safety and efficacy endpoints, but rather specific hypotheses on mechanisms of efficacy.^5^ The cell type should be carefully selected and fully characterized in terms of viability, function, optimal dose, and timing of administration.^5^ This knowledge could then also be applied in cell-enhancement strategies. A systematic analysis of the cell secretome could further profit to the translation of cell-free approaches. Besides cell-free techniques, the next-generation approaches in the cell therapy evolution might include the combinatorial cell delivery concept,^138^ the repeated sequential administration of cells,^197^ and the use of modified cells for enhanced repair.^143^ Regardless of the cell type, the main challenges of cell therapy are still the overall poor cell retention and high degree of cell death after transplantation. Until these problems are overcome, the full potential of cell therapy will likely never be realized.
